# Supplementary material for: The role of the working memory storage component in a random-like series generation
Source: PLoS One. 2024 Jan 19;19(1):e0296731. doi: 10.1371/journal.pone.0296731 (PMC10798477; doi:10.1371/journal.pone.0296731)
Supplement: S1 File — (DOCX) [file pone.0296731.s001.docx]

**Supplementary Materials for**

**“The role of the working memory storage component in a random-like series generation”**

All datasets and accompanying code are in the Open Science Framework repository: https://osf.io/ck78n/?view_only=5f48e96b3e0a45f8aa2d905330820b8c

**Information about how sample size was determined for each study and a discussion of statistical power**

As described in the method section, our primary analyses in Study 1 consist of the Generalized Additive Mixed Model and bivariate correlation. In Study 1, we collected data on algorithmic complexity and randomness judgment. (N = 151). We tested hypotheses regarding the effect of experimental conditions (visibility of the past choices in the task that required multiple thought experiments of tossing a fair coin) on the dynamic of the performance in a random series generation task. As such, we believe that the most conservative calculation of the statistical power consists of Power Analysis with Crossed Random Effects (Westfall et al., 2014). We used an online tool created by Westfall et al. (2014) with settings for Participations-for-Conditions. In Study 1, participants were exposed to 120 stimuli (on each appearance they were asked to decide whether in a coin-tossing thought experiment they got heads or tails). Therefore, assuming a moderate effect size (.3), the required N to achieve .8 power is 151. Our final sample size falls 2 participants short of reaching the minimum required sample size. That is because we employed very restrictive data exclusion criteria (described in the method section) and from the initial sample of 197 participants we excluded 47. Consequently, our sample size allows for detecting the difference between experimental conditions with .798 power.

Moreover, as described in the method section for both studies, the rest of the analyses consist of bivariate correlations. In Study 1 we collected results on the algorithmic complexity and randomness judgment (N = 151) while in Study 2 on the algorithmic complexity and the working memory capacity (N = 142). As such, we believe that the most conservative calculation of statistical power consists of a simple correlation coefficient between either the overall algorithmic complexity and randomness judgment (Study 1) and the overall algorithmic complexity and the partial span score for complex recall (Study 2). Assuming a moderate effect size (.3), the required N to achieve .8 power is 67. This calculation was done using G*Power 3.1 software with settings for Exact Test: Bivariate Correlation (Faul et al., 2007). In short, in both Study 1 and Study 2, we had sufficient power to detect whether randomness judgment and the length of the working memory capacity predict the randomness of human-generated data.

**Data exclusions and variable selection.**

The data exclusions criteria are described in detail in the body of the manuscript under the Participation subsection. In general, we used as an excluding criteria either completion time or objective measures that signaled low engagement or attention to the tasks.

We report all variables collected for the purpose of the current research and all conditions.

**Bibliography**

Faul, F., Erdfelder, E., Lang, A.-G., & Buchner, A. (2007). G*Power 3: A flexible statistical power analysis program for the social, behavioral, and biomedical sciences. *Behavior Research Methods*, *39*(2), 175–191. https://doi.org/10.3758/BF03193146

Westfall, J., Kenny, D. A., & Judd, C. M. (2014). Statistical power and optimal design in experiments in which samples of participants respond to samples of stimuli. *Journal of Experimental Psychology: General*, *143*, 2020–2045. https://doi.org/10.1037/xge0000014
