# Supplementary material for: The role of the working memory storage component in a random-like series generation
Source: PLoS One. 2024 Jan 19;19(1):e0296731. doi: 10.1371/journal.pone.0296731 (PMC10798477; doi:10.1371/journal.pone.0296731)
Supplement: Attachment — Submitted filename: Response_to_Reviewers.docx [file pone.0296731.s003.docx]

Dear Iftikhar Ahmed Khan,

We would like to thank you and the reviewers for the insightful comments and suggestions for the manuscript. We believe that they lead us to necessary improvements in our work. Below, you will find point-by-point replies to the reviewers’ comments. Original comments are in boldface, while our responses are in regular typeface.

**Journal Requirements:**

**Please review your reference list to ensure that it is complete and correct. If you have cited papers that have been retracted, please include the rationale for doing so in the manuscript text, or remove these references and replace them with relevant current references. Any changes to the reference list should be mentioned in the rebuttal letter that accompanies your revised manuscript. If you need to cite a retracted article, indicate the article’s retracted status in the References list and also include a citation and full reference for the retraction notice.**

We revised the reference list and it is complete and correct.

**Reviewer #1: I'm satisfied that the revisions address the issues and questions raised in the first round of reviews. I recommend accepting this revised version of the manuscript for publication.**

Thank you.

**Reviewer #2: I have two more questions.**

**1. in page 10/22, line 384-385, the author mentioned that Specifically, reducing cognitive load on the working memory storage 384 component led to a slower decrease in performance (see Fig. 3). However in fig3, it is the relationship between the algorithmic complexity and the time step not the performance and the time, what is the reason?**

Thank you for pointing this out. Indeed, the abovementioned sentence could have been misleading. By “performance” we meant performance in the random-like series generation task which we measured with algorithmic complexity. We added this detail in the corrected version of the manuscript.

**2. the generation of random series in the study is based on the binary series, as we know, the working memory can be tested with other protocols for example the n-back experiment. so why the author choose this experimental protocol?**

We decided to use a task based on the complex span tasks procedure developed by Turner and Engle (1989) for the measurement of working memory capacity because the structure of the task was similar to the Biesaga et al. (2021) random-like series generation model. Complex span tasks demand serial recall, whereby participants retrieve items using only self-generated cues (Kane et al., 2007). Biesaga et al.'s (2021) model also assumes sequential updating of elements highlighted in the working memory storage component without external cues. On the other hand, for example, n-back tasks require recognition, whereby participants discriminate target items from familiar foils (Kane et al., 2007). Therefore, we found a measure based on complex span tasks as more ecologically valid for testing the relationship between working memory capacity and the ability to generate random-like series.

However, certainly, in future research, it would be of utmost interest to further explore this relationship using different working memory capacity measures. Especially, as the scientific literature provides the evidence that for example complex span tasks and n-back tasks might measure different aspects of the working memory capacity (i.e., Schmiedek et al., 2014; Scharinger et al., 2017).

Biesaga, M., Talaga, S., & Nowak, A. (2021). The Effect of Context and Individual Differences in Human-Generated Randomness. *Cognitive Science*, *45*(12), e13072. <https://doi.org/10.1111/cogs.13072>

Kane, M., Conway, A., Miura, T., & Colflesh, G. (2007). Working Memory, Attention Control, and the N-Back Task: A Question of Construct Validity. *Journal of Experimental Psychology. Learning, Memory, and Cognition*, *33*, 615–622. https://doi.org/10.1037/0278-7393.33.3.615

Scharinger, C., Soutschek, A., Schubert, T., & Gerjets, P. (2017). Comparison of the Working Memory Load in N-Back and Working Memory Span Tasks by Means of EEG Frequency Band Power and P300 Amplitude. *Frontiers in Human Neuroscience*, *11*. https://www.frontiersin.org/articles/10.3389/fnhum.2017.00006

Schmiedek, F., Lövdén, M., & Lindenberger, U. (2014). A task is a task is a task: Putting complex span, n-back, and other working memory indicators in psychometric context. *Frontiers in Psychology*, *5*. https://www.frontiersin.org/articles/10.3389/fpsyg.2014.01475

Turner, M. L., & Engle, R. W. (1989). Is working memory capacity task dependent? *Journal of Memory and Language*, *28*(2), 127–154. https://doi.org/10.1016/0749-596X(89)90040-5

**3. There are some works also related to working memory and the analysis of working memory is based on neural activity within the brain. The author can have a look at these.**

**T. Xu, J. Huang, Z. Pei, J. Chen, J. Li, A. Bezerianos, N. Thakor, and H. Wang, "The Effect of Multiple Factors on Working Memory Capacities: Aging, Task Difficulty, and Training," IEEE Transactions on Biomedical Engineering, vol. 70, no. 6, pp. 1967-1978, 2023, doi: 10.1109/TBME.2022.3232849.**

**Z. Pei, H. Wang, A. Bezerianos, and J. Li, "EEG-Based Multi-Class Workload Identification Using Feature Fusion and Selection," IEEE Transactions on Instrumentation and Measurement, vol. PP, pp. 1-1, 08/27 2020, doi: 10.1109/TIM.2020.3019849.**

**Z. Pei, T. Xu, A. Bezerianos, J. Li, Y. Sun, and H. Wang, The Effect of Longitudinal Training on Working Memory Capacities: An Exploratory EEG Study. 2020.**

Thank you for bringing these publications to our attention. In future studies, we plan on using EEG to further investigate the usage of working memory in the process of random-like series generation.
